# Cyberchondria and smartphone addiction: A correlation survey among undergraduate medical students in Egypt

**DOI:** 10.1186/s42506-024-00154-y

**Published:** 2024-04-03

**Authors:** Safaa M. El-Zoghby, Nancy M. Zaghloul, Ayat M. Tawfik, Noura M. Elsherbiny, Shaimaa A. Shehata, Enayat M. Soltan

**Affiliations:** 1 Department of Family Medicine, Faculty of Medicine, Suez Canal University, Ismailia, Egypt; 2 Department of Forensic Medicine and Clinical Toxicology, Misr University for Science and Technology, Cairo, Egypt; 3 Department of Public Health and Community Medicine, Faculty of Medicine, Port-Said University, Port Said, Egypt; 4 Department of Public Health and Community Medicine, Faculty of Medicine, Suez Canal University, Ismailia, 41522 Egypt; 5 Department of Forensic Medicine and Clinical Toxicology, Faculty of Medicine, Suez Canal University, Ismailia, 41522 Egypt

**Keywords:** Smartphone use disorder, Cyberchondria, Medical students, Egypt

## Abstract

**Background:**

There is an increase in smartphone usage globally. Cyberchondria (CYB) is characterized by excessive Internet searches for health information. Smartphone addiction is constantly rising among medical students together with CYB as a collateral emerging risk, yet there is limited research available on the topic.

**Methods:**

This cross-sectional study explores the rising phenomenon of smartphone addiction and its potential role in CYB among medical students in seven Egyptian universities. A total of 1435 medical students participated in completing online questionnaires that assessed smartphone addiction and CYB using the Smartphone Addiction Scale-Short Version (SAS-SV) and Cyberchondria Severity Scale (CSS-12). Multivariable regression analysis was applied to assess predictors of smartphone addiction and CYB.

**Results:**

Based on the used scales, 57.6% of students were suffering from smartphone addiction, and 85.8% exhibited a moderate degree of CYB. There was a significant positive correlation between smartphone addiction scores and CYB (*p* < 0.05). The multivariable regression models revealed that four factors including using Facebook, using WhatsApp, increasing time spent on the Internet per day, and a higher CYB score increase the probability of smartphone addiction. The factors that increase the probability of CYB are using Facebook, an increase in the frequency of Internet searches, a higher degree of worry about one’s health or a family member’s health, and being a smartphone addict.

**Conclusion:**

Smartphone addiction among undergraduate medical students is prevalent. Social media use, time spent online, and smartphone addiction were linked with the risk of CYB. Regular physical activity decreases the probability of smartphone addiction. Awareness programs and increased mental and physical activities are required to help reduce smartphone addiction among youth.

## Introduction

The rapid development of technology, particularly smartphones with various Internet-connected applications, has led to increased smartphone usage in our modern lives [1]. This rise in technology development is accompanied by a collateral increase in smartphone use. The Organization for Economic Cooperation and Development (OECD) reported that the average time spent on the Internet by individuals aged 14 to 24 has reached approximately 4.5 h per day [2]. This excessive use has given rise to the concept of “smartphone addiction,” which exhibits characteristics of a behavioral addiction, causing psychological, social, and physical problems [3, 4]. Although smartphone addiction is not yet defined as such by the *Diagnostic and Statistical Manual 5* (DSM-5) or in the International Classification of Diseases 11 (ICD-11) draft, the DSM-5 mentioned nondrug addiction, such as Internet and smartphones as a concern that needs to be discussed for possible future inclusion [5]. Additionally, the integration of smartphones and the Internet into everyday activities, including remote learning and seeking health information, has become more prevalent [6, 7]. Approximately, 4.5 billion people worldwide in 2019 used the Internet to search for medical information. In the United States, a study revealed that approximately 88% of users search the Internet for information on health-related issues [8].

Hypochondriasis, also known as illness anxiety disorder (IAD), is a psychiatric condition characterized by excessive worry or fear about having a serious medical illness, despite little or no evidence of medical pathology. Individuals with hypochondriasis often misinterpret bodily sensations or minor symptoms as indicative of a severe medical condition, leading to heightened anxiety and distress [9]. This preoccupation with health concerns may cause significant impairment in daily functioning, as the individual may seek frequent medical consultations, undergo numerous unnecessary medical tests, and experience significant distress and impairment in their overall quality of life. The term “hypochondriasis” has been replaced with “illness anxiety disorder” in the fifth edition of the *Diagnostic and Statistical Manual of Mental Disorders* (DSM-5) to better reflect the nature of the condition and to reduce the stigma associated with the term “hypochondriasis” [9].

However, the rise of searching for health information online has also led to the emergence of “cyberchondria.” Cyberchondria is a recent term that is illustrated as the anxiety state about one’s health due to the repetitive and excessive behavior of obtaining online health information, leading to anxiety and compulsive behaviors and hindering daily activities [10–12]. Cyberchondria can result in various negative effects, such as fear, stress, depressive symptoms, and an increased risk of anxiety disorders [7]. It may also lead to an economic burden due to repeated consultations with different physicians [13]. Extravagant use of smartphones, easy accessibility to the Internet, higher health anxiety, and lack of trusted sources of information are all risk factors for cyberchondria [7, 12]. Technology has positively impacted the medical field, with smartphones and the Internet being valuable tools for medical students’ learning process [14]. Medical students are continuously exposed to medical information, which could subject them to a high prevalence of health anxiety [15]. Furthermore, it was found that medical students have a higher prevalence of Internet addiction than the general population [16], with evidence showing that addiction is associated with a greater likelihood of health anxiety and cyberchondria [17].

Although previous studies have investigated smartphone addiction among medical students in Egypt, cyberchondria and the relationship between it and smartphone addiction have not been thoroughly studied. Given the limited information about the prevalence of cyberchondria in Egypt, this study was conducted to investigate the prevalence of cyberchondria and the relationship between it and smartphone addiction among undergraduate medical students in Egypt.

## Methods

### Study design and setting

This was a cross-sectional study that assessed smartphone addiction and CYB among a sample of undergraduate medical students in Egypt. This study was conducted from March 2022 to June 2022 by using an anonymous online questionnaire. Medical students were recruited from seven Egyptian universities: Suez Canal, Port Said, Suez, Misr University for Science and Technology, Helwan, South Valley, and Alexandria universities.

### Sample size

The sample size was calculated using the Epi Info CDC software calculator https://www.cdc.gov/epiinfo/index.html based on a study that estimated the prevalence of CYB in Pakistan [18]. The required sample size was 362 participants, and by assuming a non-response rate of 10%, the total required sample size was 398 participants. The level of confidence is set at 95% with a *p*-value significance below 0.05. However, the final sample size was 1435 participants in the current study as students were excited and willing to participate on a large scale. A larger sample size increases the statistical power of the study, reduces the risk of type II errors, provides more precise estimates, and narrows the confidence intervals around the study findings.

### Sampling technique

The snowball convenience sampling technique was used. Data was collected using an online Google Form that was shared on different social media groups (WhatsApp, Facebook, & Telegram groups) used by faculty staff to communicate with their students. These channels are used to get in contact with the students throughout the academic year for follow-up or any announcements, including questionnaires used for research purposes. Upon receiving the link, information regarding the research and its aim appeared on the first page with informed consent at the end, and then questions appeared consecutively. It took about 5–7 min to complete the questionnaire. Inclusion criteria included undergraduate students (enrolled in one of the participating institutions at the time of the study) of both sexes, aged 18 or more, who consented to participate.

### Data collection tools

A structured self-administrated questionnaire was used, and the questionnaire consisted of three parts:

- *First part: Sociodemographic and smartphone data*, which included questions about the participant’s gender, residence, academic year, academic grades, physical activity, and chronic illnesses among them and their families, in addition to information about smartphone use, for instance, time spent using smartphones and Internet, its frequency, different reasons for use, frequency of searching health-related information, and kinds of used applications.
- *Second part*: Smartphone Addiction Scale- Short Version (SAS-SV) — SAS-SV is a validated scale that was developed by a South Korean research team in the English language as a short version of the original SAS. It consists of 10 items that assess daily-life disturbance, signs of withdrawal, cyberspace-oriented relationships, overuse, and tolerance. Each item is scored using a Likert-type scale that ranges from 1 (strongly disagree) to 6 (strongly agree) which is later translated into a total score that ranges from 10 to 60, with cut-off scores of more than 31 in males and more than 33 in females indicating Smartphone addiction [19, 20]. The SAS-SV scale has been validated in a different population [19] and found to have good reliability (Cronbach’s alpha range from 0.79 to 0.91) [20]. The English version was used as the participants were medical students. No adaptation to the Egyptian culture was performed.
- *Third part*: Cyberchondria Severity Scale (CSS-12) — This is a 12-item self-reported tool that was modified by McElroy et al. in 2019 [21] — based on the original 33-item scale — to assess the CYB severity. The 12 items assess CYB factors of (1) *compulsion*, which implies interference with different aspects of a person’s life (for example, researching symptoms or perceived medical conditions online interrupts my work); (2) *distress*, which indicates negative emotional responses affected by online health research (OHR) (e.g., I start to panic when I read online that a symptom I have is found in a rare/serious condition); (3) *excessiveness*, refers to repetition and consuming time (e.g., I enter the same symptoms into a web search on more than one occasion); and (4) *reassurance*, which points out to the degree where OHR gives rise to consulting a physician [22].

Each item was scored using a Likert-type scale ranging from 1 “never” to 5 “always,” which was then summed to form a total score that ranged from 12 to 60. If scores fall at or below the 25th percentile, it indicates a low level of CYB, and if scores lie between the 25th and the 75th percentiles, it indicates a moderate level of CYB, and scores that fall at or above the 75th percentile indicate a high level of CYB. The CSS-12 has been validated in different populations and showed a good reliability (Cronbach’s α for its subscales ranging between 0.73 and 0.90) [21–23].

### Statistical analysis

Data were coded and entered into Microsoft Excel (2019 version), which was later exported for analysis using IBM Statistical Package for Social Sciences (SPSS) software (version 23) [24]. Descriptive statistics were presented by frequencies, percentages (%), and mean ± SD. Pearson correlation was used to assess associations between quantitative variables. The chi-square test was used to test the statistical significance of categorical data. Multivariable regression analysis was used to assess predictors of smartphone addiction and CYB. *p*-value < 0.05 was considered statistically significant based on the level of confidence of 95%. All independent factors were added to the regression model.

### Ethical considerations

Obtaining ethical approval from the ethics committee was done, and informed consent was included at the beginning of the Google Form questionnaire and was obtained by clicking the agree button before filling out the questionnaire for all participants. Those who were less than 18 years old were excluded. All the collected data was anonymous and confidential; only the research team had access to the research data, and they were responsible for all aspects of data collection and analysis without external partners. Participation was voluntary, and they could withdraw from the study without stating any reason.

## Results

### Sociodemographic data of the studied participants

A total of 1435 participants were included in the study. The mean age of participants was 21 ± 2 years, 51.4% were females, and 68.5% were from urban residences. About 48.2% were fourth academic-year students, and 96.1% were single. The latest grade point average (GPA) was B among 42.2% and A among 34.7% of the participants (Table 1).

**Table 1:** Sociodemographic data of the studied sample of medical students in Egyptian universities (*N* = 1435)

Sociodemographic data	
**Age (years)**	
* Mean* ± *SD*	21 ± 2
**Gender**	
* Male*	698 (48.6)
* Female*	737 (51.4)
**Residence**	
* Rural*	452 (31.5)
* Urban*	983 (68.5)
**Academic year**	
* First*	207 (14.4)
* Second*	55 (3.8)
* Third*	404 (28.2)
* Fourth*	691 (48.2)
* Fifth*	27 (1.9)
* Sixth*	51 (3.61)
**Marital status**	
* Single*	1379 (96.1)
* Married*	7 (0.5)
* Engaged*	49 (3.4)
**Last year’s grade point average (GPA)**	
* A*	498 (34.7)
* B*	605 (42.2)
* C*	163 (11. 3)
* D*	11 (0.8)
* E*	0 (0)
* First year, no GPA*	158 (11)

About 10.3% of the participants suffered from chronic illness; the most frequently reported one was autoimmune diseases (16.9%). Regular physical activity was reported by 36.7% of the students.

### Internet usage and health-related information

Regarding the use of the Internet, 45.5% of the students spent 6 to 12 h per day browsing different Internet websites, where the most frequently reported websites were Facebook (65.2%), WhatsApp (61.6%), YouTube (48%), and Google (30.5%), respectively. Nearly a quarter of the students (25.3%) reported searching health-related information for personal use (not study) almost every day, and 45.9% of them reported always being worried about their own health or a family member’s health (Table 2).

**Table 2:** Searching for health-related information and use of Internet data of the studied sample of medical students in Egyptian universities (*N* = 1435)

Health-related information and use of Internet data	*n* (%)
**Chronic illness**	
* Diabetes mellitus*	12 (8.1)
* Hypertension*	11 (7.4)
* Cardiac*	6 (4)
* Renal*	2 (1.4)
* Autoimmune*	25 (16.9)
* Absent*	1287 (89.7)
**Regular physical activity**	
* Yes*	526 (36.7)
* No*	909 (63.3)
***Number of hours that they spend on different Internet websites per day***	
* Less than 6 h*	703 (49)
* From 6 to 12 h*	653 (45.5)
* More than 12 h*	79 (5.5)
**The most commonly used programs**^**a**^ **(select all possible)**	
* Facebook*	935 (65.2)
* WhatsApp*	884 (61.6)
* Online shopping*	129 (9)
* Google*	438 (30.5)
* Twitter*	244 (17)
* YouTube*	689 (48)
* Snapchat*	149 (10.4)
* Instagram*	147 (10.2)
* Others*	212 (14.8)
**Frequency of searching health-related information for personal use (not study)**	
*(Nearly) everyday*	363 (26.2)
*4 to 5 days a week*	183 (13.1)
*2 to 3 days a week*	287 (20.7)
*About once a week*	244 (17.6)
*Less often*	310 (22.4)
**Degree of worry about one’s health or family members’ health**	
*Always*	658 (45.9)
*Usually*	413 (28.8)
*Sometimes*	301 (21)
*Rarely*	47 (3.3)
*Never*	16 (1)

^a^The total does not add to 100% because of multiple responses

### Cyberchondria and smartphone addiction scales results

Cyberchondria Severity Scale (CSS) showed a mean total score of 32.2 ± 9. More than three-quarters of the students (85.8%) suffered from a moderate degree of CYB, while the mean total score of the smartphone addiction scale was 34.3 ± 12, with more than half of students (57.6%) suffering from smartphone addiction.

There was a positive statistically significant correlation between the mean smartphone addiction score and mean CYB score along its four sub-scale scores (*p* < 0.05). There was also a statistically significant association between being a smartphone addict and having moderate-to-high degree of CYB (*p* < 0.05) (Table 3).

**Table 3:** Associations between cyberchondria and smartphone addiction among the studied sample of medical students in Egyptian universities (*N* = 1435)

**Descriptive statistics of Cyberchondria Severity Scale and Smartphone Addiction Scale**					
		**No. (%)**			
**Cyberchondria Severity Scale** *Mean (SD)*	*Compulsion*	7.5 (3)			
	*Distress*	7.7 (3)			
	*Excessiveness*	9.2 (3)			
	*Reassurance*	7.8 (3)			
	***Total score***	32.2 (9)			
**Cyberchondria categories** *Frequency (%)*	*Low < 25th*	55 (3.8)			
	*Moderate 25th–75th*	1231 (85.8)			
	*High ˃ 75th*	149 (10.4)			
**Smartphone Addiction Scale** *Mean (SD)*	***Total score***	34.3 (12)			
**Smartphone addiction categories** *Frequency (%)*	*Present*	827 (57.6)			
	*Absent*	608 (42.4)			
**Analytic statistics of Cyberchondria Severity Scale and Smartphone Addiction Scale**					
**Correlations**	**Smartphone Addiction Score**				
	***R***	***p*-value**			
**Compulsion**	0.234	0.0001*			
**Distress**	0.257	0.0001*			
**Excessiveness**	0.292	0.0001*			
**Reassurance**	0.191	0.0001*			
**Total cyberchondria score**	0.296	0.0001*			
**Associations**: *Frequency (%)*		**Cyberchondria**			
		**Low**	**Moderate**	**High**	***p*-value**
**Smartphone Addiction Scale**	**Addict**	21 (38.2)	692 (56.2)	114 (76.5)	< 0.05**
	**Non-addict**	34 (61.8)	539 (43.8)	35 (23.5)	

^*^Correlation is significant at the 0.01 level (2-tailed)

**Statistically significant, chi-square test

Regarding the multivariate regression analysis for the possible predictors of smartphone addiction, using Facebook, and WhatsApp, spending more time on the Internet per day, and being cyberchondriac were statistically significant predictors (*p* < 0.05), while physical activity was significantly decreasing the probability of smartphone addiction (Table 4). Regarding other variables such as age, gender, academic year, and residency, none of them was a statistically significant predictor of smartphone addiction.

**Table 4:** Regression analysis of predictors of smartphone addiction among the studied sample of university students in Egypt (*N* = 1435)

Independent variables	B	*p*-value	Exp. (B)	95% CI for exp. (B)	
				**Lower**	**Upper**
**Age**	.036	0.434	1.037	0.947	1.135
**Gender**	− 0.147	0.215	0.863	0.684	1.089
**Residence**	− 0.151	0.222	0.860	0.675	1.095
**Academic year**	− .028	0.703	0.972	0.841	1.124
**Marital status**	− .035	0.820	0.966	0.714	1.305
**GPA**	.051	0.498	1.052	0.908	1.219
**University**	.030	0.348	1.030	0.968	1.096
**Facebook**	0.526	.0001*	1.693	1.330	2.154
**WhatsApp**	0.296	.019*	1.344	1.049	1.722
**Online shopping**	− 0.272	0.185	0.762	0.509	1.139
**Google**	− .063	0.645	0.939	0.717	1.228
**Twitter**	.094	0.546	1.099	0.809	1.492
**YouTube**	.029	0.815	1.029	0.808	1.311
**Snapchat**	.022	0.908	1.023	0.700	1.494
**Instagram**	.013	0.945	1.013	0.704	1.458
**Other programs**	0.247	0.129	1.280	0.930	1.762
**Chronic illness**	0.241	0.201	1.272	0.880	1.839
**Physical activity**	− 0.439	.0001*	0.645	0.510	0.816
**Frequency of searching health-related information**	− .059	0.105	0.942	0.877	1.012
**Hours they spend on Internet per day**	0.438	.0001*	1.550	1.280	1.876
**Degree of worry about one’s health or family members’ health**	− .096	0.100	0.909	0.811	1.018
**Cyberchondria score**	0.929	.0001*	2.533	1.827	3.513

^*^Statistically significant

The multivariate regression analysis for the possible predictors of cyberchondria level shows that the frequency of searching health-related information, degree of worry about one’s health or family member’s health, and being addicted to smartphones were statistically significant predictors (*p* < 0.05) (Table 5).

**Table 5:** Regression analysis of predictors to cyberchondria among the studied sample of university students in Egypt (*N* = 1435)

Variables	Unstandardized coefficients		Standardized coefficients	*t*	*p*-value	95.0% confidence interval for B	
	**B**	**Std. error**	**Beta**			**Lower bound**	**Upper bound**
**Age**	− 0.173	0.317	− .019	− 0.544	0.587	− 0.795	0.450
**Gender**	0.984	0.815	.031	1.207	0.228	− 0.615	2.582
**Residence**	1.380	0.861	.041	1.602	0.109	− 0.309	3.069
**Academic year**	− 0.167	0.512	− .012	− 0.326	0.745	− 1.170	0.837
**Marital status**	1.102	1.089	.026	1.012	0.312	− 1.035	3.239
**GPA**	− 0.307	0.525	− .017	− 0.584	0.559	− 1.337	0.724
**University**	0.163	0.222	.020	0.732	0.464	− 0.273	0.599
**Chronic illness**	0.895	1.306	.017	0.685	0.493	− 1.667	3.456
**Physical activity**	0.491	0.851	.015	0.577	0.564	− 1.178	2.160
**Frequency of searching health-related information**	0.916	0.257	.090	3.568	.0001*	0.412	1.419
**Hours spent on the Internet per day**	− 0.196	.680	− .007	− 0.289	0.773	− 1.531	1.138
**Degree of worry about one’s health or family members’ health**	1.650	0.407	0.103	4.057	.0001*	0.852	2.447
**Smartphone addiction**	9.871	0.818	0.309	12.069	.0001*	8.266	11.475

^*^Statistically significant

## Discussion

Nowadays, there is concern over smartphone addiction as a public health issue, particularly among college students, a lot of key questions concerning CYB remain vague. The present study was designed to investigate smartphone addiction, and CYB, and assess how smartphone use affects CYB among medical students in Egypt. The study revealed that a considerable percentage of the students (45.5%) spend 6 to 12 h using smartphones per day. In line with the present study, a study among medical students in Mansoura University, Egypt, reported that 65.8% of their participants spent less than 8 h/day using smartphones, and 66.8% of the participants used smartphones for social interaction [25]. These findings are congruent with the pattern of smartphone usage in other studies, such as the study conducted in the Kingdom of Saudi Arabia where 55.8% of students reported using smartphones for more than 5 h per day [26], while it was higher than the rate in Malaysia where 65.9% of students reported using smartphones for more than 3 h daily [27].

Moreover, we found that more than half of students (57.6%) have smartphone addiction according to the questionnaire cut-off points. This result aligns with rates among Egyptians mentioned in other studies, such as the study among medical students at Mansoura University [25] where they reported the presence of smartphone addiction in 53.6% of students. However, a higher rate (74.7%) of smartphone addiction was found among medical students at Suez Canal University, Egypt [28]. In other Arab countries such as Jordan, the presence of smartphone addiction was 59.8% among students [29], while in Lebanon a rate of 49% was reported [30]. Internationally, a study among Chinese students reported smartphone addiction in 52.8% of their sample [31], while a much higher rate was found in India in around 85% of medical college students in the Andaman and Nicobar Islands [32].

While smartphones provide students the opportunity to continue learning and studying remotely, their use among university students is usually more of a psychosocial one, primarily observed among individuals who are often immature and of low self-regulatory mechanisms, so they are more likely to succumb to excessive use not only in spare time but also during class [33]. Moreover, youth in Arab countries are particularly vulnerable as they are mesmerized by the modern technology imported from developed ones, which may explain the higher rate of addiction among them [26, 28, 30, 31]. Lower rates were reported in other countries such as Iran 9.3% [34] and Belarus 10% [35]. These lower rates could be explained by the conservative nature of some countries, such as Iran, where some restrictions could be found on Internet and smartphone use. The lower rate in Belarus may be related to the time of conducting the study, as it was done more than 10 years ago.

As regards CYB, about a quarter of our students (25.3%) reported searching for health-related information nearly every day, whereas 45.9% of them also reported being always worried about one’s health or a family member’s health. The mean total score of the Cyberchondria Severity Scale (CSS) was 32.2 ± 9, where more than three-quarters of the students (85.8%) scored a moderate degree of CYB. Similar to the age group in our study, a Lebanese study used the CSS-12 to measure the severity of cyberchondria among 449 Lebanese University students aged around 24 years, but 70.6% of the sample were females (in our study, it was nearly half). The comparison of latent mean scores revealed no significant differences across genders, in either the cyberchondria total score or its facets. The CSS-12 score was positively associated with anxiety (convergent validity), OCD, and stress. Previous studies revealed a link between high eHealth literacy and cyberchondria, which could be explained by the fact that eHealth literacy is directly proportional to Internet addiction [12, 36, 37]. Another explanation for the high CYB rate is that frequent searching of the Internet for health concerns may lead to a healthy lifestyle, or they may be able to discuss and assist a physician in diagnosis and management during the appointment [38]. Because cell phones nowadays are widely available and provide fast and easy access to health data, smartphone addiction is a key contributing factor to CYB.

Moreover, we reported the presence of a good, positive, and statistically significant correlation between smartphone addiction score and CYB across its four sub-scales scores which agreed with Köse and Murat who also found a similar result using the Spearman’s rank correlation coefficient analysis [7]. Recently, electronic health websites offered free and easy access to gather information, which made the repeated behavior of browsing the Internet feel comfortable regarding individual health concerns. Although CYB associates online searching for health-related information with reducing worry or anxiety, excessive browsing sometimes leads to additional psychological distress due to rare complications or misconceptions [10, 12]. Another possible explanation of CYB is that instead of “physician shopping,” which is the strong urge to visit numerous doctors for the same issue, individuals alternatively search the Internet to avoid the additional costs [13]. Additionally, the COVID-19 pandemic boosted the frequency of Internet searches for health-related issues [39].

In the present study, it was found that the increased frequency of Internet searches increases the probability of CYB. These findings disagree with Köse et al.’s study [7] which reported that no relationship was found between CYB levels and time spent online. This could be explained by looking at the reasons for using smartphones in the first place, and the time planned for Internet use is crucial for medical students to complete their assignments, projects, and research papers.

In congruence with the present study, findings by Ivanova in Bulgaria regarding the degree of worry about one’s health and CYB showed that individuals who were extremely worried about their health had a significantly higher mean score on CSS and its subscale. Internet addiction was positively correlated with escalations and persistence of health concerns as indicators of CYB, and health anxiety was also correlated with escalation and persistence of concerns [40]. Also, a systematic review by McMullan et al. found a statistically significant association between being addicted to a smartphone and having a moderate or high degree of CYB [14].

The concept of seeking online health information has a strong positive correlation with the frequency of searching the Internet for information. Previous studies proved that CYB is linked to smartphone addiction [41, 42]. An individual who suffers from CYB-related behaviors will use the Internet to gather relevant health information for reassurance. However, if a person did not reach the wanted or unpredictable answers, he would enter a “vicious circle,” as the nature of CYB suggests, and he would end up with higher levels of anxiety with information overload [43]. Thus, the need for repeated reassurance leads to uncontrolled smartphone usage, which consequently leads to excessive smartphone addiction [41].

The current study may help medical professionals and psychologists deal with medical students who have cyberchondria by gaining an understanding that their Internet searches for health-related information simply exacerbate their anxiety; hence, the psychologist should implement cognitive behavioral therapy to lessen health anxiety.

### Limitations

The use of a cross-sectional design limits the inference of causal association between cyberchondria and Internet addiction. The study population was primarily Egyptian medical students which might limit the generalizability of the results to nonmedical students in other universities and the general population. Moreover, data collection was done through a self-administered questionnaire which may result in reporting bias.

## Conclusions

Our findings revealed that smartphone addiction increases the probability of CYB, while regular physical activity decreases the probability of smartphone addiction. Promoting mental health among medical students through educational programs helps lessen smartphone addiction. Enhancing physical activity will, in turn, help control the level of CYB in undergraduate students and ultimately decrease their level of smartphone addiction. More research is needed in Egypt to understand cyberchondria better and develop preventative strategies.

## Abbreviations

Apps: Applications
CSS-12: Cyberchondria Severity Scale
CYB: Cyberchondria
DSM-5: *Diagnostic and Statistical Manual 5*
ICD-11: The International Classification of Diseases 11
OECD: The Organization for Economic Co-operation and Development
SAS-SV: Smartphone Addiction Scale-Short Version
